# Integrating spatial transcriptomics with single-cell transcriptomics reveals a spatiotemporal gene landscape of the human developing kidney

**DOI:** 10.1186/s13578-022-00801-x

**Published:** 2022-06-03

**Authors:** Hongwei Wu, Fanna Liu, Yu Shangguan, Yane Yang, Wei Shi, Wenlong Hu, Zhipeng Zeng, Nan Hu, Xinzhou Zhang, Berthold Hocher, Donge Tang, Lianghong Yin, Yong Dai

**Affiliations:** 1 Clinical Medical Research Center, Guangdong Provincial Engineering Research Center of Autoimmune Disease Precision Medicine, Shenzhen Engineering Research Center of Autoimmune Disease, The Second Clinical Medical College of Jinan University, Shenzhen People’s Hospital, Shenzhen, 518020 Guangdong China; 2 Institute of Nephrology and Blood Purification, The First Affiliated Hospital of Jinan University, Jinan University, Guangzhou, 510632 China; 3 Shenzhen Far East Women & Children Hospital, Shenzhen, 518000 Guangdong China; 4 Department of Medicine Nephrology, Medical Faculty, Mannheim Heidelberg University, 68167 Mannheim, Germany; 5 Guangxi Key Laboratory of Metabolic Disease Research, Central Laboratory of Guilin NO. 924 Hospital, Guilin, 541002 China

**Keywords:** Spatial transcriptomics, Single-cell transcriptomics, Spatiotemporal gene landscape, Human kidney development

## Abstract

**Background:**

Research on spatiotemporal gene landscape can provide insights into the spatial characteristics of human kidney development and facilitate kidney organoid cultivation. Here, we profiled the spatiotemporal gene programs of the human embryonic kidneys at 9 and 18 post-conception weeks (PCW) by integrating the application of microarray-based spatial transcriptomics and single-cell transcriptomics.

**Results:**

We mapped transcriptomic signatures of scRNA-seq cell types upon the 9 and 18 PCW kidney sections based on cell-type deconvolution and multimodal intersection analyses, depicting a spatial landscape of developing cell subpopulations. We established the gene characteristics in the medullary regions and revealed a strong mitochondrial oxidative phosphorylation and glycolysis activity in the deeper medullary region. We also built a regulatory network centered on *GDNF-ETV4* for nephrogenic niche development based on the weighted gene co-expression network analysis and highlighted the key roles of *Wnt*, *FGF*, and *JAG1-Notch2* signaling in maintaining renal branching morphogenesis.

**Conclusions:**

Our findings obtained by this spatiotemporal gene program are expected to improve the current understanding of kidney development.

**Supplementary information:**

The online version contains supplementary material available at 10.1186/s13578-022-00801-x.

## Introduction

Human kidney development is a dynamic process controlled by complex cell-to-cell communication and molecular signal pathways. Anatomically, the two major kidney regions, the cortical region where the filtrate is yielded and the medullary region where urine is concentrated, take charge of different biological functions. The dominating infectious challenge derives from bacteria ascending the ureter into the kidney pelvis [1]. In mammals, the nephron and collecting system, which originate entirely from the intermediate mesoderm, show diverse developmental histories. During embryogenesis, the epithelial ureteric bud (UB) subdivides progressively into ramifications through multiple transcription factors modulation (*Wt1*, *Pax2*, *Lim1*, *Sall1*, *Eya1*) and ultimately generates the entire collecting system, including the ureter and collecting ducts [2]. Around the UB tips, nephron progenitor cells (NPCs) polymerize to form the cap mesenchyme (CM) and further differentiate into pretubular aggregate (PTA), renal vesicle (RV), s-shaped body (SSB), podocyte, proximal tubules, and loops of Henle through sophisticated mesenchymal–epithelial transition [2, 3].

Recently, the single-cell transcriptome makes possible the in-depth investigation in transcriptional heterogeneity and regulatory mechanism among cell subsets in the developing kidney, providing novel insights into the pathophysiological mechanism for acquired renal disease progression [3–5]. These studies, however, fall short of capturing the spatial location information of each cell, thus limiting our understanding of specific functions performed by various spatial regions of the kidney, especially spatial heterogeneity of the identical cell type. For instance, urine concentration capacity is perturbed along kidney tissue’s depth, which may be particularly relevant for the medullary circulation. However, the mechanism of renal medullary endothelium adapts to dehydration and the molecular heterogeneity of renal endothelial cells from the cortex, glomeruli, and medulla remains unknown. Therefore, resolving the spatial composition of cellular subpopulations in the kidney is a top priority. The cutting-edge spatial transcriptomics (ST) technology enables researchers to describe an unbiased map of transcripts over an entire tissue section using spatially barcoded oligo-deoxythymidine microarrays, which has preliminarily been utilized in studies of human fetal heart development and tumor immune microenvironment [6–9]. To reveal the unbiased spatial organization of kidney cell populations and to assess spatiotemporal gene expression dynamics during human fetal kidney development, we integrate microarray-based spatial transcriptomics with single-cell RNA-seq on fetal kidney tissues at 9 and 18 weeks using the 10× Genomics platform, giving new perspectives on robust targets for regenerative medicine and kidney organoid cultivation.

## Materials and methods

### Slide preparation, fixation, staining, and imaging

We performed microarray-based spatial transcriptomics on human fetal kidneys from 9 to 18 post-conception weeks (PCW), equivalent to 7 and 16 weeks of embryonic development, respectively. This study was conducted in accordance with the principles of the Declaration of Helsinki and was authorized by the ethics board of Shenzhen People’s Hospital (LL-KY-2019591). Spatial transcriptomics slides included four identical 6.2 × 6.6 mm capture areas, each with approximately 5000 unique gene expression spots (10× Genomics) as previously described [6]. Each spot was covered with approximately 2 × 10^8^ oligonucleotides for mRNA capture, containing a spot-unique 18-mer spatial barcode, a randomized 7-mer unique molecular identifier (UMI), and a poly-20(T)VN. Fresh tissues were washed with cold PBS and frozen by isopentane (2-methybutane Sigma) and liquid nitrogen bath. The frozen tissues were then embedded in an optimal cutting temperature (OCT) compound, cryosectioned at a thickness of 10 μm, and mounted onto the Visium Tissue Optimization Slides (3000394, 10× Genomics) and Visium Spatial Gene Expression Slides (2000233, 10× Genomics). Next, the sectioned slides were warmed to 37 °C for 1 min and fixed in 36.5% formaldehyde (#F8775, Sigma-Aldrich) diluted 1:10 in 1× PBS (#09-9400, Medicago) for 10 min, followed by washing in 1× PBS. The tissues were then dehydrated with isopropanol for 1 min and subjected to H&E staining. Further, the slides were mounted with 80% glycerol, and brightfield histology images were taken using a 10× objective (Plan APO) under a Nikon Eclipse Ti2-E inverted fluorescent (27,755 × 50,783 pixels for TO, 13,332 × 13,332 pixels for GEX).

### Tissue permeabilization, reverse transcription, and probe release

For pre-permeabilization, the sections were incubated with 0.2 mg/mL BSA and 200 units of collagenase diluted in HBSS buffer at 37 °C for 20 min. Tissues were next permeabilized at 42 °C for 4 min in 0.1% pepsin in HCl. Then, 100 µL of 0.1× SSC buffer was used to wash the wells post-incubation. Reverse transcription (RT) Master Mix containing reagents [1× First-strand buffer (Invitrogen), 0.5 mM of each dNTP, 5 mM dithiothreitol, 0.2 µg/µL BSA, 1% dimethylsulfoxide, 50 ng/µL Actinomycin D, 2 U/µL RNaseOUT (Invitrogen) and 20 U/µL Superscript III (Invitrogen)] was added to the permeabilized tissue section, followed by overnight incubation at 42 °C. Tissues were digested away from the slide with 1% 2-mercaptoethanol in RLT buffer (Qiagen^®^, Valencia, CA, USA) with interval shaking, followed by incubation at 56 °C for 1 h with proteinase K (Qiagen) diluted 1:8 in PKD buffer (Qiagen). The slides were further sequentially rinsed in SSC buffer with different concentrations (2 × SSC for 10 min at 50 °C; 0.2 × SSC for 1 min at 25 °C; 0.1 × SSC for 1 min at 25 °C). Next, the slides were imaged to visualize the incorporated fluorescent nucleotides, together creating a cDNA footprint as previously detailed [10]. Here, we selected 6 min as the optimal permeabilization time based on the effect of the fluorescent print.

### Spatial library preparation and sequencing

The spatially barcoded cDNA was released by incubating arrays with release mix (8.75 µM of each dNTP, 0.1 U/µL USER enzyme, 0.2 µg/µL BSA). After incubation, the supernatants with cleaved probes were collected, and the released cDNA was then converted into dsDNA. Next, this dsDNA was purified using beads and underwent in vitro transcription overnight (1× T7 Enzyme Mix, 1× T7 Reaction Buffer, 7.5 mM of each NTP, and 1 U/µL SUPERaseIN). The remaining purified cDNA was indexed as follows: 98 °C for 3 min, 25 cycles of 98 °C for 20 s, and 72 °C for 5 min. The Agilent 2100 Bioanalyzer™ (Agilent Technologies, Santa Clara, CA, USA) and a Qubit dsDNA HS Assay Kit (Life Technologies, Carlsbad, CA, USA) were utilized for the library purification and quantification following the manufacturer’s instructions [6]. Next, paired-end sequencing was performed on the Illumina HiSeq3000 system (Illumina, San Diego, CA, USA) with a sequencing depth of 250–270 M read-pairs per sample. Quality control analysis of pre-processing data was performed using FastQC.

### ST raw data annotation, filtering, and processing

The Space Ranger Pipeline (10X Genomics, Pleasanton, CA, USA) was used to conduct data quality statistical analyses of the ST sequencing data. Reads were mapped to the human GRCh38 genome assembly and aligned using STAR v.2.5.1b [11]. Spots with more than 10% mitochondrial genes or fewer than 200 detected gene counts were discarded. We used the SCTransform function of the Seurat package (version 3.1.3) in R to normalize the gene-spot matrices and further screened hypervariable genes using the FindVariableGenes function. Dimensionality reduction was performed on the 30 most significant components as determined by the PCElbowPlot function, followed by ST clusters identification using the FindClusters function (settings: reduction, type = “pca”, resolution = 0.9). Visualization of ST clusters was finally carried out using Uniform Manifold Approximation and Projection (UMAP) or t-Distributed Stochastic Neighbor Embedding (TSNE) in the Seurat package.

### Multimodal intersection analysis (MIA) and cell-type deconvolution analysis

We downloaded online scRNA-seq datasets of fetal kidneys from 9 to 18 post-conception weeks (Accession number: GSE114530). The data processing and statistical analysis were performed as Hochane et al. described [4]. After merging similar clusters, we identified 15 cell types. Subsequently, we screened sets of respective genes in the scRNA-seq data by comparing gene expression in each cell type with the genes expressed in the remaining cell type. Genes with significantly higher expression (adjusted P-value < 0.05, |log2 FC| > 0.25) were identified as the cell-type-specific gene sets for subsequent analysis. Similarly, we identified tissue region-specific gene sets in each spatial subpopulation in the ST data. After extracting representative gene sets, we applied MIA to analyze the overlap between each pair of ST and scRNA-seq gene sets and compute a hypergeometric test to evaluate significant enrichment [6]. We applied Seurat to transfer cluster labels from scRNA-Seq to spatial transcriptomic spots. For deconvolution analyses, a SPOTlight neural network analysis was performed as Elosua-Bayes et al. described [12]. The proportion of signature of each scRNA-seq cell type in renal cortex, medulla, and pelvis was calculated as follows: the sum of the proportions of each cell type in a different region is calculated separately, and then divided by the sum of the proportions of that cell type across all spots. Only cell types that contribute at least 10% to the spot signature are calculated.

### Weighted gene co-expression network analysis (WGCNA)

An overview of the WGCNA methodology and data processing is as previously described [13]. Briefly, we screened differential expressed gene sets (adjusted P-value < 0.05, |log2 FC| > 0.25) in the 15 ST cell clusters and constructed a complex gene co-expression network. Subsequently, WGCNA identified essential modules which were consisted of highly interconnected genes using unsupervised clustering. The gene expression levels in each module were summarized by the first principal component (the module eigengene) and were applied to evaluate whether modules are associated with special kidney regions. To describe the specific characteristics of each cell subpopulation in the fetal kidney, we identified biologically interesting modules associated with spatial region based on module-region relationship (*P* < 0.01 and r > 0.4). A weighted correlation network was carried out and key driver genes or transcription factors in the interesting modules were determined, which depended on the sum of the neighbor edge weights of a node in the network.

### Differential gene expression and functional enrichment analyses

Differential genes of individual ST clusters against all other ST clusters were identified using pair-wise comparison analysis with the FindAllMarkers function (settings: logfc.threshold = 0.1, test.use="bimod”, min.pct = 0.01). GO characteristics and KEGG enrichment of targeted gene sets were calculated using the clusterProfiler package (version 3.8.1). Pearson correlation across different ST clusters matrix was performed by gplots package.

## Results

### Spatial transcriptomics of fetal kidney tissues

**Fig. 1:**
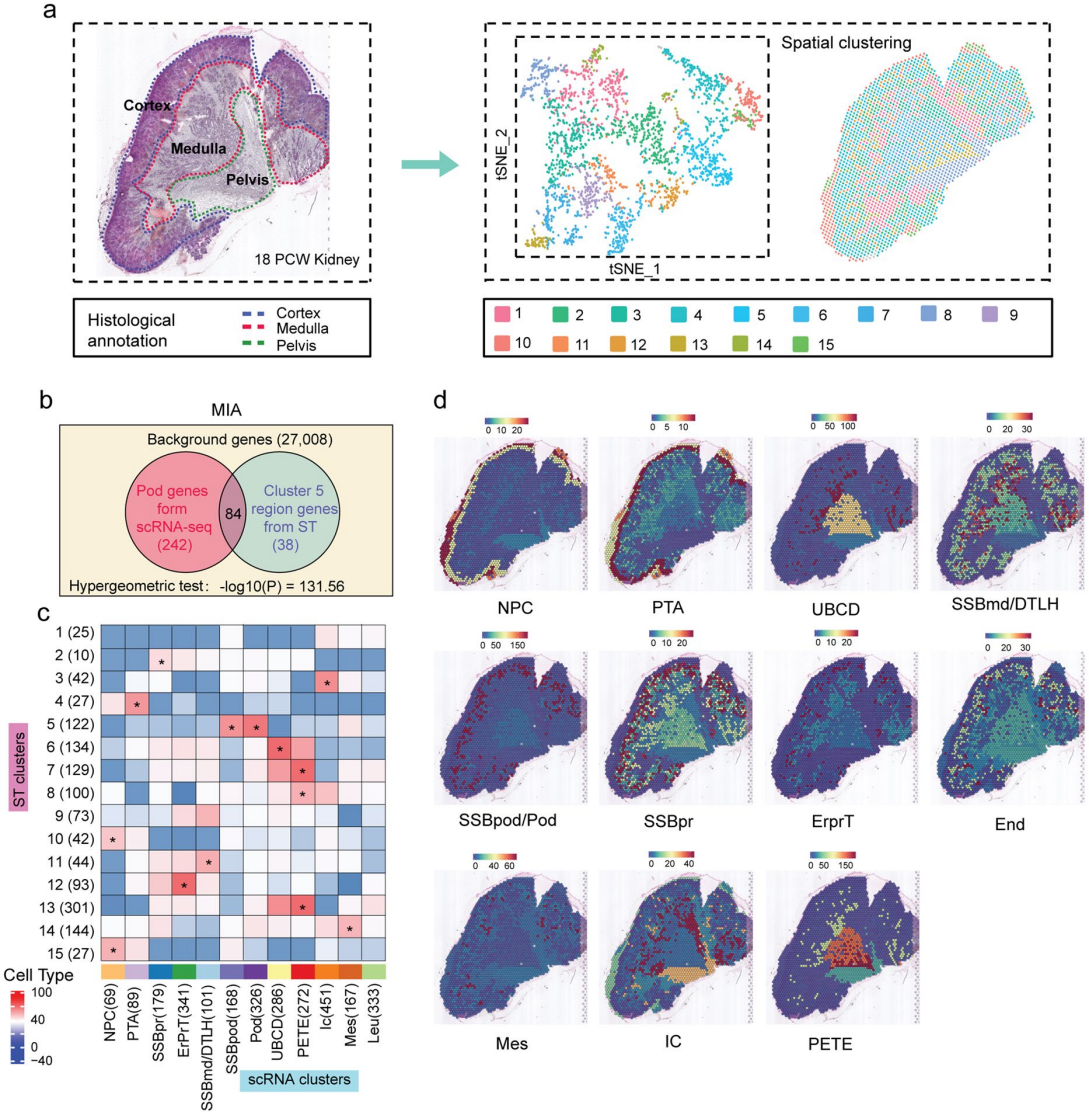
Spatial cell-type annotation in the human embryonic kidney from 18 weeks post-conception. **a** A schematic diagram displaying the artificial division of the three compartments (cortex, medulla, and pelvis regions) of the kidney based on histomorphology. t-SNE shows the cell types identified with all the single cells, and colors indicate the 15 spatial clustering assignments; **b** Multimodal intersection analysis (MIA). The hypergeometric test was applied to evaluate the significant intersection of the respective genes specifically identified in a given cell type (podocyte in this case) and region-specific gene sets (see “Materials and methods” section). **c** MIA map of all scRNA-seq-identified cell types and ST-defined clusters. Each element in the matrix was computed as described in (**b**) for all pairs of cell types and tissue regions using the same 27,008 background genes. The bar on the bottom indicates – log10 (*P*-value) calculated by hypergeometric test. *Represents the closest relationship between scRNA-identified cell types and the ST-defined clusters (*P* < 0.01). **d** Space projection of scRNA-identified cell types on the kidney tissue from 18 weeks post-conception. Scale bar: the blue to red gradient corresponds to the matching degree between cell-type-specific (scRNA-seq) and region-specific (ST-seq) gene sets. PCW, post-conception weeks; ICs, interstitial cells; PTA, pretubular aggregate; UBCD, ureteric bud/collecting duct; SSBmd/DTLH, distal tubule/loop of Henle and s-shaped body medial precursor cell; NPC, nephron progenitor cell; SSBpr, s-shaped body proximal precursor cell; ErprT, early proximal tubule. PETE, Pelvic segment transitional epithelium; Mes, mesangial cell; Pod, podocyte

To delineate the unbiased spatial transcriptomics profile of the fetal kidney, we mounted cryosections of the embryonic kidneys at two post-conception time points (9 PCW and 18 PCW) on the spatially barcoded ST microarray slides. Based on the hematoxylin and eosin staining and brightfield imaging results obtained, we annotated the 18 PCW kidney tissue for three distinct anatomical regions: the cortical region, where podocytes, proximal tubule cells, and part of the distal tubule cells were detected; the medullary region, which contained principally loop of Henle cells and collecting duct cells; and the pelvic region, where the pelvic epithelium and the transitional epithelium of the ureter accounted for the main part [14] (Fig. 1a). After DNA synthesis, ST library construction, and data sequencing, we detected 2697 spots under the 18 PCW kidney section at a median depth of 8554 UMIs, including 3626 genes per spot. At 9 PCW, the two sections of the kidney contained a total of 175 spots, including 1486 genes per spot (Additional file 1: Fig. S1a, b and Additional file 2: Table S1).

### Spatial mapping of cell type subpopulations across tissue regions

We clustered the ST spots by performing principal component analysis and then projected each spot onto the kidney section based on spot-unique spatial barcodes. These ST spots clustered into 15 spatially conserved cell subpopulations (Fig. 1a). To determine the identity of these ST cell clusters, we integrated the ST and scRNA-seq datasets using multimodal intersection analysis (MIA) developed by Moncada et al. (see “Materials and methods” section). We identified 15 cell subsets of the 18 PCW kidney tissue based on the scRNA-seq data (Additional file 1: Fig. S1c, d), and then calculated the overlap between cell type-specific and region-specific gene sets. For instance, the gene set identified in the spatial region of ST cluster 5 overlapped significantly with podocyte-specific genes of the scRNA-seq data (Fig. 1b). Therefore, we labeled ST cluster 5 as podocytes. Similarly, ST clusters 1 and 3 were identified as interstitial cells (ICs), cluster 4 as pretubular aggregate (PTA), cluster 6 as ureteric bud/collecting duct (UBCD), clusters 7, 8, and 13 as Pelvic segment transitional epithelium (PETE), clusters 9 and 11 as distal tubule/loop of Henle and s-shaped body medial precursor cells (SSBmd/DTLH), clusters 10 and 15 as nephron progenitor cells (NPCs), cluster 2 as s-shaped body proximal precursor cells (SSBpr), cluster 12 as early proximal tubule (ErprT), and cluster 14 as mesangial cells (Mes) (Fig. 1c). We also used specific cell-type gene markers known from the literature to perform manual annotation, and the annotation result was consistent with the result obtained from MIA (Additional file 1: Fig. S1e). We mapped transcriptomic signatures of scRNA-seq clusters directly upon the 18 PCW kidney section, for the first time, to depict a spatial landscape of developing cell subpopulations (Fig. 1d).

### Cell-type deconvolution of ST-seq cell clusters

**Fig. 2:**
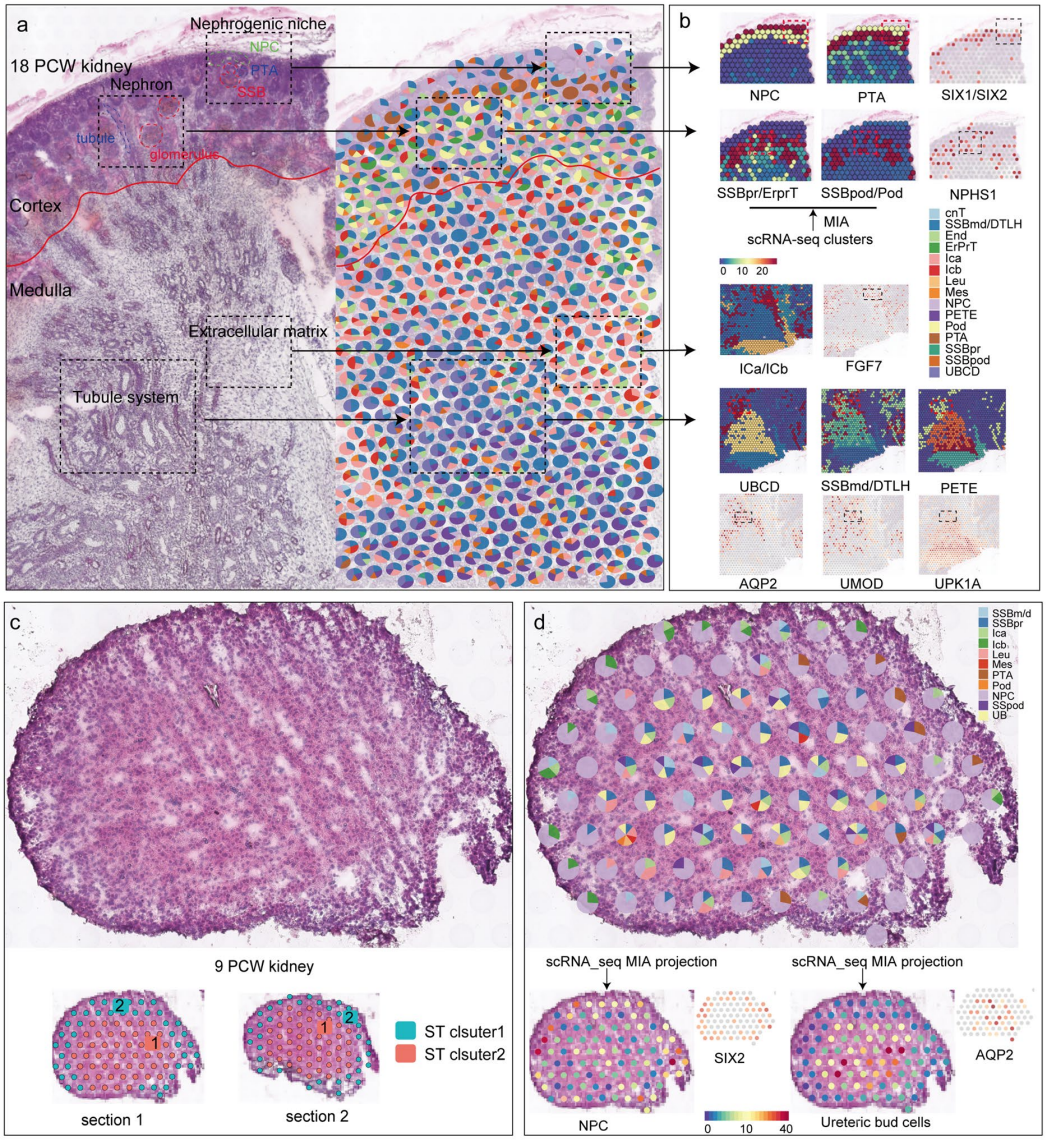
Cell-type deconvolution of the kidney tissues from 9 and 18 weeks post-conception. **a** The histological structure of the 18 PCW kidney and the proportion of signatures of different scRNA-seq cell types in each ST spot. Each pie chart represents the contribution of scRNA-seq cell types to the transcriptomic signature of each ST spot. Only cell types that contribute at least 10% to the spot signature are shown. Dashed boxes at the same level corresponding to the same histology section location. The junction between the cortex and the medulla is marked with a red line. **b** Spatial mapping of scRNA-seq cell subsets performed by MIA, and expression localization of marker genes. The red box corresponds to the histology section location selected by the dashed box in (**a**). **c** Spatial spots identification for the human embryonic kidneys from 9 post-conception weeks. **d** The proportion of signatures of different scRNA-seq cell types in each ST spot (up) and the spatial mapping of scRNA-seq cell subsets performed by MIA (down)

Considering the potential overlap of multiple cell types in each spot, we sought to apply Spotlight analysis to define the contribution of each scRNA-seq cluster to the expression profile of each ST spot. After Seurat deconvolution, we obtained the proportion of signatures of different scRNA-seq cell types in each spot (Fig. 2a and Additional file 1: Fig. S2a, b). These spots were largely overlaid upon the expected histological features. For example, NPC and PTA cell clusters contributed the greatest proportion of signature to spots overlying the nephrogenic niche (the outer cortex), while the podocyte, ErprT, and endothelial cell clusters localized over the spots overlying the nephron region (the inner cortex). In the medullary region, the interstitial cells dominated the spots mapping to the extracellular matrix region, while the UBCD and DTLH cell clusters dominated the spots in the tubule region. These deconvolution results were consistent with the spatial mapping of scRNA-seq cell subsets performed by MIA (Fig. 2b). For instance, the transcriptomic signature of NPC and PTA and their corresponding marker genes (SIX1 and SIX2) were consistently mapped to the nephrogenic niche region. Notably, we discovered some SSB structures (histology) and a proportion of SSBm/d cells (deconvolution method) in the nephrogenic nice. However, the SSBm/d cluster failed to map to this histological location (MIA method), suggesting that cell-type deconvolution could provide more specific information on the spatial localization of scRNA-seq cell subpopulations.

Spatial transcriptomic spots from the 9 PCW kidney were clustered into two individual cell subpopulations (Fig. 2c). We identified 12 scRNA-seq cell subsets (Additional file 1: Fig. S1c) and used the MIA method to depict a spatial projection of these cell subpopulations on the 9 PCW kidney section. The gene set identified in ST cluster 1 overlapped significantly with the scRNA-seq UB and SSB-specific genes, while ST cluster 2 mainly matched the expression profiles of NPC and IC cells (Additional file 1: Fig. S2c). We ran a deconvolution analysis and calculated the proportion of each scRNA-seq cluster in each ST spot. The result showed that NPC and IC cell clusters contributed the greatest proportion of signatures to the spots assigned to ST cluster 2, while NPC, UB, and SSB (SSBpr and SSBm/d) dominated the spots assigned to ST cluster 1 (Fig. 2d and Additional file 1: Fig. S2c). The identification results were in general agreement between the two methods.

We next compared the spatial characteristics of each cell subset across 9 PCW, 18 PCW, and adult human kidneys to depict dynamic changes in the proportions and spatial locations of these cell subpopulations during kidney development. In the 9 PCW kidney, the cells displayed a striated distribution with no apparent histological structures and partitions (Fig. 2c). We calculated the proportion of each scRNA-seq cell subpopulation arising from each spot and observed a large fraction of renal precursor cells such as NPC, UB, and SSB cells (Additional file 1: Fig. S2d). Interestingly, NPC, usually accompanied by IC cells, was principally localized around the peripheral region, whereas UB and SSB cells were restricted to the central region. This cellular distribution characteristic might underlie the formation of cortical and medullary regions (Fig. 2d). In the 18 PCW kidney, in addition to the numerous renal precursor cells (NPC, PTA, SSB, and UB), we also discovered a progressive increase in the number of mature cell types (Pod, Mes, cnT, CD, DTLH) (Additional file 1: Fig. S3d). Since the expression profile of DTLH resembled that of the SSBm/d (precursor cells of DTLH), we classified them as the SSBmd/DTLH cluster. In this developing period, histological partitioning of the kidney tissue was already evident. To better characterize the spatial location of developing cell subpopulations, we divided the 18 PCW kidney section into three regions (cortex, medulla, and pelvis) based on the histological feature (Fig. 1a). We extracted 1011, 779, and 839 ST spots from the cortex, medulla, and pelvis areas, respectively. Spots were only counted if completely in the frame. The fractional occupancy of different scRNA-seq cell types in each spot is summarized in Additional file 3: Table S2. The results showed that cells (NPC, PTA, SSBpr, SSBpod, Mes, ErprT, End, and Pod) associated with the development of the nephron system were largely positioned in the cortical region, while cells (UBCD, ICb) associated with the development of collecting system were mainly localized in the medullary region (Additional file 1: Fig. S3e). Equal proportions of distal tubules and loop of Henle cells could be observed in both cortical and medullary regions. The spatial distribution of mature cell subpopulations (Mes, ErprT, Pod, cnT, DCT, LOH) at 18 PCW kidney was almost same as that in the adult kidney [15], except that progenitor cells of the adult kidney have disappeared (Additional file 1: Fig. S3f).

### The spatial heterogeneity of the renal cortex and medulla

**Fig. 3:**
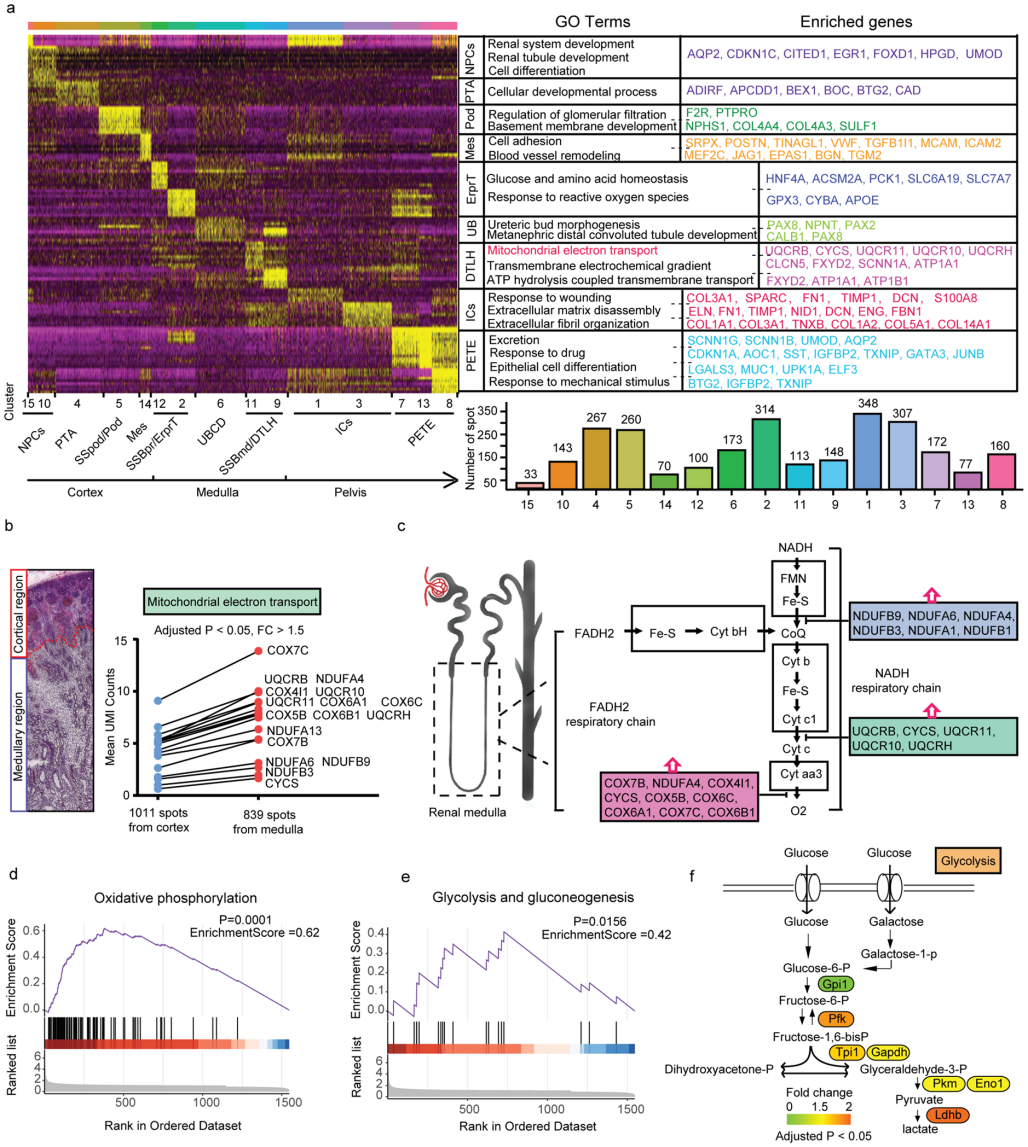
Biological function identification in each spatial cell type and the spatial gene characteristics of the medullary region. **a** Left, heatmap displaying the differentially expressed genes that were cell type specific. Right, the enriched biological processes of each ST cell type and the enrichment of the corresponding genes. The histogram on the bottom shows the number of ST spots identified in each spatial cluster; **b** Identification of high variable genes in the medullary region. ST spots were extracted from the medulla and the remaining regions (cortex and pelvis), respectively, and the mean UMI counts of each gene were calculated. The red circles represent the differential UMI counts of the genes related to mitochondrial electron transport in the medulla, compared with the remaining regions; **c** Schematic diagram shows the process of NADH respiratory chain electron transport and its regulatory genes based on gene ontology analysis. Gene set enrichment analysis showing the significant changes in oxidative stress (**d**) and glycolysis pathway (**e**) in the medullary region. *P <* 10^− 2^ and enrichment score > 0.5 denote high enrichment; **f** Pathway map showing the changes in the transcript levels of glycolysis-related genes in the medullary region. Scale bar: the yellow and orange colors represent upregulated genes (adjusted P-value < 0.05, |log2 FC| > 0.25), green color indicates genes change that did not reach the fold change threshold

We identified 2280 up-regulated genes among these 15 ST clusters using Seurat analysis. Gene ontology analysis showed that the enrichment function of each cell subset was consistent with the relative biological processes, as also previously reported [14] (Fig. 3a and Additional file 4: Table S3). For example, descending thin limb of Henle (DTLH) cells were chiefly involved in ion transmembrane transport and electrochemical gradient maintenance, while podocytes regulated mainly glomerular filtration [16, 17]. To explore the spatial heterogeneity of the renal cortex and medulla, we extracted ST spots in the cortical and medullary regions, respectively (as described above). Compared to the cortical area, the medullary region had dramatically overexpressed genes associated with ion transport, and unexpectedly high enrichment of the genes associated with mitochondrial electron transport (adjusted P < 0.05, |log2 FC| > 0.25 ) (Fig. 3b and c). Gene set enrichment analyses (GSEA) showed that variable genes highly expressed in the medullary region were involved predominantly in the biological processes of the nicotinamide adenine dinucleotide (NADH) respiratory chain electron transport, oxidative phosphorylation (OXPHOS) (*NDUFB*, *NDUFA*, *UQCRB*, *CYCS*, *COX7B*, *COX7C*), and glycolysis (*PFK*, *TPI1*, *GAPDH*, *PKM*, *LDHB*) [18, 19] (Fig. 3c–f). These findings suggested a strong activity of mitochondrial ATP synthesis and utilization in the medullary region, reflecting the different energy metabolism requirements of cells in different regions of the developing kidney. Energy metabolism plays a pivotal role in determining whether a cell differentiates, proliferates, or maintains quiescent. Therefore, we depicted the energy metabolic feature of each cell subset based on the scRNA-seq data. The results revealed that SSBmd/DTLH, SSBpr/ErprT, and PTA highly expressed glycolysis- and OXPHOS-related genes (Additional file 1: Fig. S3a, b), while NPC cells showed low expression of energy-related genes. Interestingly, metabolic energy requirements did not correlate exclusively with cell proliferation. For example, SSBmd/DTLH cells overexpressed energy metabolism-related genes but remained in a low proliferative state (Additional file 1: Fig. S3c). We believe that this phenomenon can be ascribed to the heterogeneity of the spatial structure of the renal tissue. The renal cortex comprised mostly the human nephrogenic niche (NPC and PTA) (Additional file 1: Fig. S3d), where PTA cells required more energy to maintain proliferation than undifferentiated NPC cells. However, there were only a few new cells generated at the level of the medulla from direct differentiation of the NPCs and UB cells, but a larger number of renal tubular cells underwent active transport of different ions, amino acids, and glucose, which required a considerable amount of energy (Additional file 1: Fig. S3e). Therefore, we speculate that PTA cells in the cortical region relied on OXPHOS and glycolysis for proliferation, while cells in the medullary region required enormous energy to fit the ion transport demand.

### Transcriptional characteristics of the medullary region

**Fig. 4:**
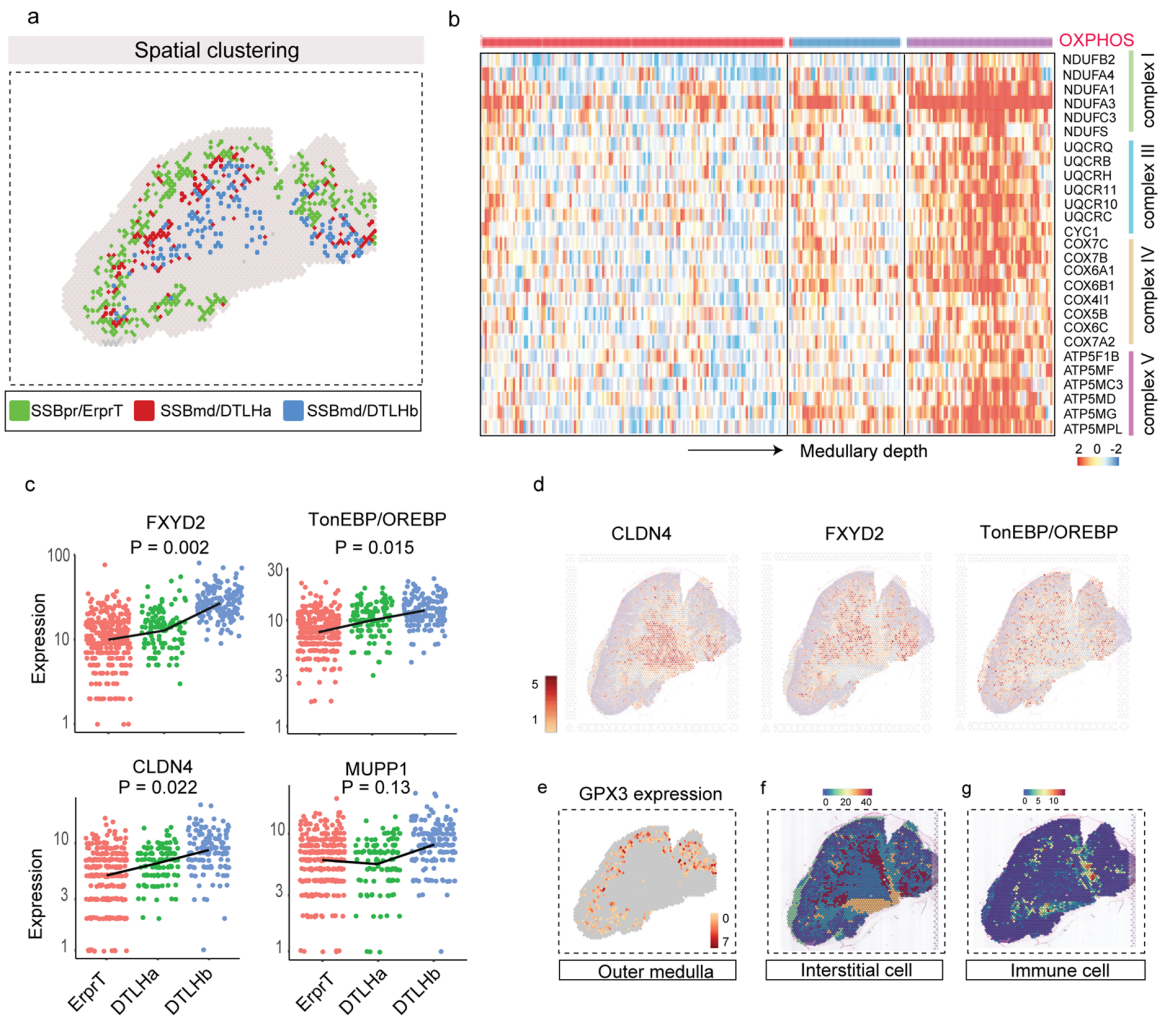
Heterogeneity analysis of the three DTLH clusters located in different renal medullary regions. **a** The spatial location of the SSBpr/ErprT and SSBmd/DTLH clusters. The green color represents the SSBpr/ErprT cluster (outer layer of the medulla), the red color indicates the SSBmd/DTLHa cluster (the middle layer of the medulla), and the blue color denotes to SSBmd/DTLHb cluster (the inner layer of the medulla); **b** Heatmap illustrating the genes encoding complex subunits of mitochondrial OXPHOS, gradually increasing from outer medulla to inner medulla; **c** Expression changes of the genes related to osmotic stress (FXYD2, TonEBP/OREBP, and MUPP1/CLDN4), gradually increasing from outer medulla to inner medulla. The black line represents the variation trend of the average gene expression. *P* values represent the overall comparison between the three groups using ANOVA. A *P*-value < 0.05 is statistically significant. **d** Spatial distribution of FXYD2, TonEBP/OREBP, and CLDN4; **e** Spatial image depicting the expression of GPX3 in the outer medulla. Scale bar: the white to red gradient corresponds to the gene upregulation levels. **f** Space projection of scRNA-identified interstitial cells. Scale bar: the blue to red gradient corresponds to the matching degree between cell- type-specific (scRNA-seq) and region-specific (ST-seq) gene sets; **g** Space projection of scRNA-identified immune cells

We observed SSBpr/ErprT and two types of SSBmd/DTLH distributed along with the spatial depth of the renal medulla (Fig. 4a). Interestingly, genes encoding complex subunits of mitochondrial OXPHOS were overexpressed along with the medullary depth (Fig. 4b), revealing that DTLH cells located in the inner medulla required higher energy to maintain metabolic activity compared to proximal tubule cells. We also found that *FXYD2*, *MUPP1/CLDN4*, and *TonEBP/OREBP* showed high expression levels in the deeper medullary region (Fig. 4c, d). These genes were essential for the adaptation of epithelial cells to medullary hyperosmolality, implying that cells in the early embryonic stage were ready to cope with the hyperosmotic environment by altering gene transcription [20–23]. Compared to the DTLH, ErprT cells in the outer medulla characteristically expressed *GPX3*, an extracellular glutathione peroxidase, suggesting a robust antioxidant defense [24, 25] (Fig. 4e). Another interesting phenomenon in the medullary region was that the distribution of leukocytes was mainly localized around interstitial cells (Fig. 4f, g). This finding provided two possible messages. First, the immune system might develop independently of the nephrogenic niche because the two clusters were spatially separated. Also, there might be a mutual dependency between the development of interstitial and immune cells.

### Spatial transcriptional network and regional function of NPCs in the cortical area

WGCNA was used to group the representative genes obtained from different kidney areas into modules with strongly covariant patterns (see “Materials and methods” section). This informative method can efficiently be used for the construction of the biological function of the module associated with specific cell subsets such as NPCs and Pods from distinct regions based on the highly specific transcript profiles and hub genes of a diverse cell subpopulation [26].

**Fig. 5:**
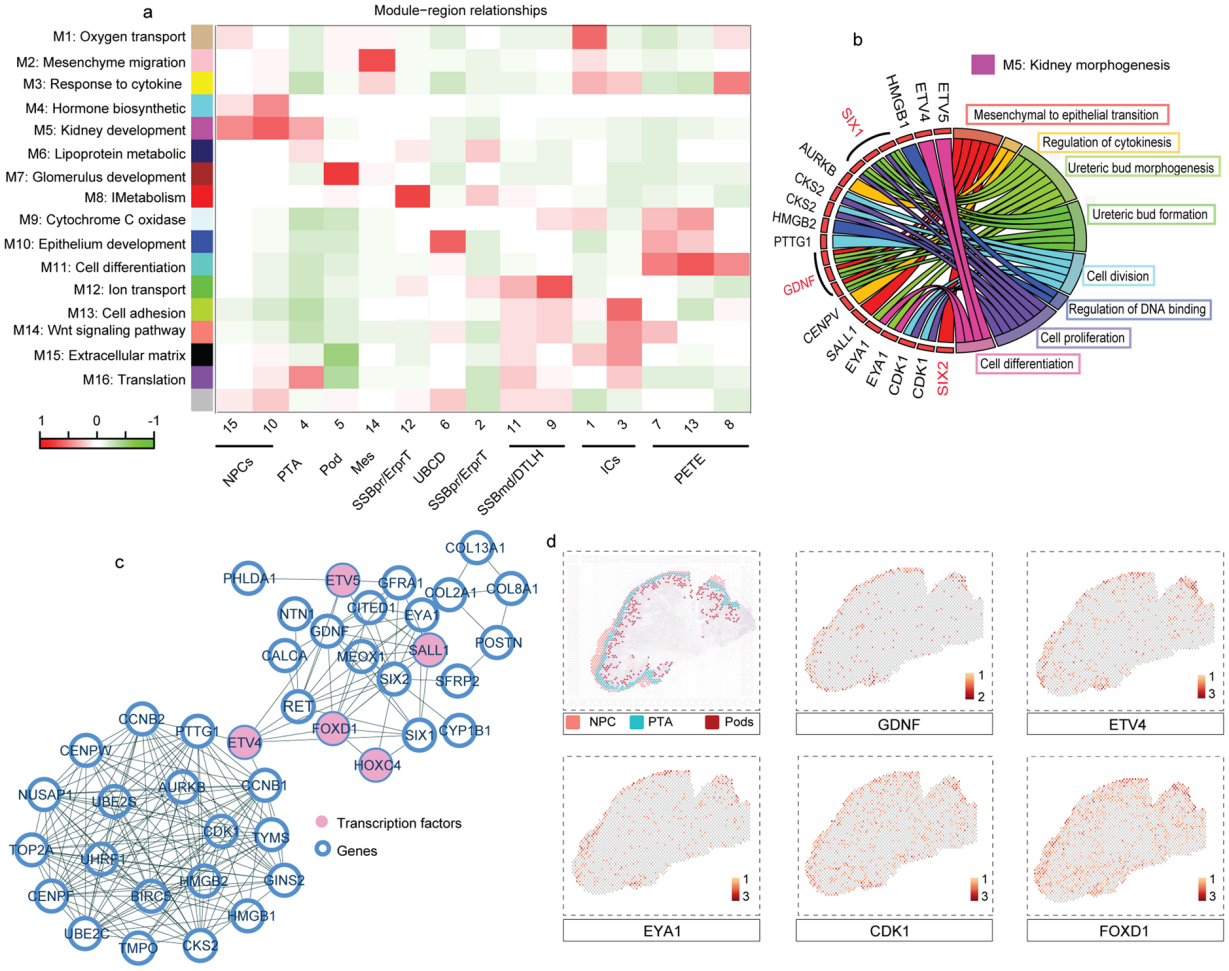
Spatial transcriptional network and heterogeneity of NPCs in the cortical regions of the **a** Correlations between module eigengenes and the spatial regions of the kidney. The numbers within the heatmap represent Pearson correlations and *P*-values (red, positively correlated; green, negatively correlated) of the module-region associations. The scale bar on the bottom indicates the Pearson correlation coefficient. R > 0.4 and a *P*-value < 10^− 3^ represent a strong correlation; **b** Gene ontology enrichment analysis reveals the biological function of differentially expressed genes in the M5 module related to kidney morphogenesis. **c** The connections among genes identified in the M5 module were constructed using online STRING analysis. The genes with an interaction score > 0.4 are shown in the network view. Pink color represents transcription factors, and blue indicates genes; **d** Spatial distribution of GDNF, ETV4, EYA1, CDK1, and FOXD1

After removing the module with unclear gene clustering (M17), we identified 16 distinct co-expressed patterns and further defined these modules based on their biological functions and key genes. The enriched terms of gene ontology for these gene modules were corresponded to related functions of the fetal renal cell subsets, as previously reported [14], such as ion transport (M8 and M12), endothelium development (M7), and epithelium development (M5 and M10) (Additional file 1: Fig. S4). The biological process of the specific gene module overlapped the function of the cell type with a high module-region relationship (r > 0.4, see “Materials and methods” section), indicating the presence of functional heterogeneity in diverse spatial regions (Fig. 5a). M5 represented the common features of NPC and PTA from diverse cortical regions, and enriched GO terms for M5 were associated with kidney morphogenesis and regulation of ureteric bud (UB) formation [27, 28] (Fig. 5a, b and Additional file 1: Fig. S4). To better determine the synergistic effect of each gene in M5, we established a co-expression network and uncovered the ‘hub’ genes based on gene connectivity. We not merely identified typical well-known genes related to nephrogenic niche development, such as *ETV4*, *EYA1*, and *GDNF* [29, 30], but also found rarely reported co-expressed genes, such as cell cycle-regulation genes *CDK1* and cell differentiation-regulation transcription factor *FOXD1* [31, 32], that had a synergistic activity with these conventional genes, thus providing more comprehensive regulatory information regarding NPC and PTA development (Fig. 5c). Notably, these co-expressed genes were almost exclusively localized to the outer cortical region (Fig. 5d). Consistent with the ST results, genes in the M5 network were also overexpressed in NPC and PTA cells in scRNA-seq data (Additional file 1: Fig. S5). In addition, we found that the ribosome-related module (M16) had a strong correlation with PTA rather than NPC (Fig. 5a), which was consistent with Hochan’s scRNA data (Additional file 1: Fig. S6), implying the action of a compensation mechanism for an enhanced protein turnover in PTA. Genes related to mitochondrial energy metabolism and ribosome synthesis were significantly upregulated in PTA, revealing an active developmental process in the deeper cortical regions.

### The function of spatial communication in cell fate determination

**Fig. 6:**
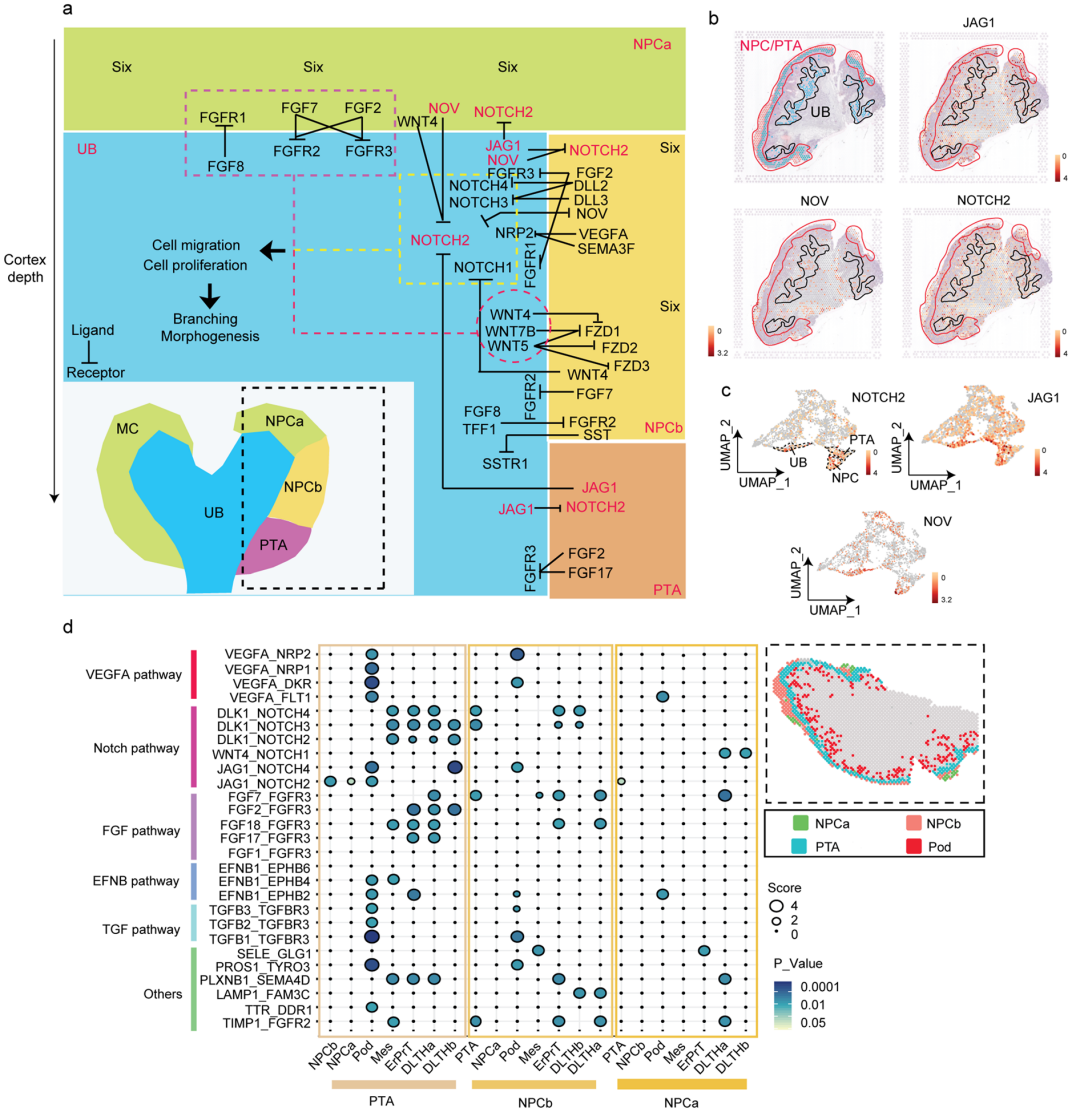
Cellular communication and spatial cross-talk in the fetal kidney. **a** Schematic diagram of the signaling transduction regulation between NPC, PTA, and UB cells. The blue region represents the UB cell cluster, the faint yellow color on the top indicates the NPCa cell cluster, the deep yellow color denotes the NPCb cell cluster, and the orange color shows the PTA cell cluster. Structure chart on the left: the bottom indicates the spatial location of NPCs, PTA, and UB clusters. The bold arrows indicate significant ligand-receptor effects (matching score > 2 and P < 10^− 2^). The most important ligand-receptor pairs controlling UB branching morphogenesis and nephron progenitor cell proliferation are marked in red; **b** Spatial location of Notch2, JAG1, and NOV. The NPC/PTA cluster is circled in red, and the UB cluster is circled in black. **c** UMAP shows the expression levels of Notch2, JAG1, and NOV in the 15 spatial cell clusters. Scale bar: the white to red gradient corresponds to the gene upregulation levels. **d** Left, cellular communication between NPC and their distal differentiated cells. The size of the circle represents the matching score. The color corresponds to the *P*-value. Right, the spatial location of the cell clusters

Cellular communication and signaling transduction regulation between NPCs and UB are the earliest process in fetal kidney development [2]. We performed ligand-receptor interaction analysis to investigate cellular communication and spatial cross-talk in the fetal kidney [33]. *GDNF/Ret* signaling is normally the predominant stimulus for UB formation and branching morphogenesis [28]. Consistent with previous studies, we found high expression of *GDNF* and its co-receptor *GFRα1* and *Ret* in NPC. In addition, transcription factors (e.g., *Pax2*, *Sall1*, and *Eya1*) required for normal *GDNF* expression were specifically expressed in NPC [34] (Additional file 1: Fig. S7a). We established signaling transduction between UB, NPC, and PTA cells. The results showed that FGF, Notch, and Wnt signals were crucial ligand-receptor pairs between UB and NPC (score > 1, P < 0.01), which were in keeping with previous reports (Fig. 6a and Additional file 1: Fig. S7b) [35–37]. We found that *Notch2* and its ligands *JAG1* and *NOV* were overexpressed in both UB and NPC/PTA clusters (Fig. 6b, c), suggesting that *JAG1/NOV-Notch2* signaling might be a pathway indispensable for the interaction between NPC/ PTA and UB cells, thereby regulating UB branch formation and NPC/PTA proliferation [38]. We then established cellular communication between each ST cluster. PTA cells had more significant signaling transduction with distal differentiated cells than NPC cells, suggesting that the closer the cell space distance increased the communication activity between these cells (Fig. 6d).

### Cell lineage differentiation and maturation pathway in developing kidneys

**Fig. 7:**
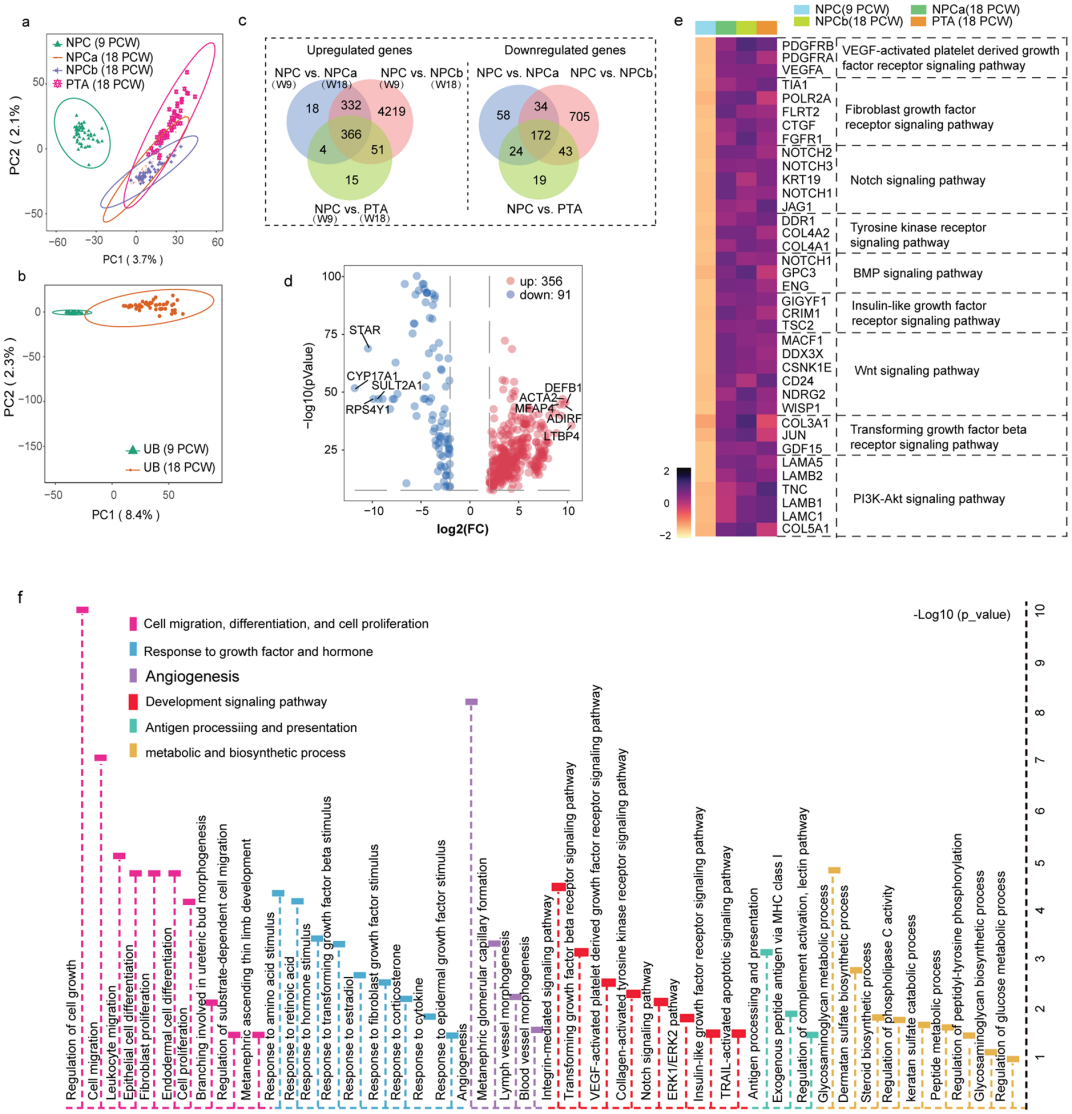
Cell lineage differentiation and maturation pathway in developing kidneys. **a** Principal component analysis showing the transcriptional similarity of the NPCs from the 9 PCW and 18 PCW kidneys; **b** Principal component analysis indicating the transcriptional similarity of UB from the 9 PCW and 18 PCW kidneys; **c** Venn diagram of the up-and down-regulated genes between the NPC cluster from the 9 PCW kidney and the NPC, PTA clusters from the 18 PCW kidney; **d** Volcano plot showing the differentially expressed genes in the NPC cluster from the 18 PCW kidney compared with the NPC cluster from the 9 PCW kidney; **e** KEGG pathway analyses of the 366 common upregulated genes in NPC/PTA from the 18 PCW kidney; **f** Changes in the biological functions and pathways during the UB development (in kidneys from 9 PCW to 18 PCW)

We used PCA to cluster each ST spot from different embryonic ages to obtain further insight into the expression profile differences between 9 and 18 PCW kidneys. We distinguished all the spots between 9 PCW and 18 PCW kidneys based on their transcriptional similarity (Fig. 7a, b). Subsequently, we focused mainly on the investigation of potential pathways associated with NPC and UB development. We identified 366 common upregulated genes in the NPC cluster of the 18 PCW kidney compared with the 9 PCW kidney (Fig. 7c, d). Some significant development pathways, including the PI3–Akt pathway, TGF-β receptor pathway, fibroblast growth factor receptor pathway, and tyrosine kinase receptor pathway, were essential for NPC development (Fig. 7e and Additional file 1: Fig. S8a). We established a wide range of complex development processes in the UB, including cell migration and differentiation, cellular response to growth factors and hormones, angiogenesis, and metabolic and biosynthetic processes (Fig. 7f). Additionally, we performed an analysis of the ligand-receptor interaction between NPCs and UB cells from the 9 PCW kidneys. High enrichment of Wnt, FGF, and Notch signaling was found between the NPC and UB cell communication (Additional file 1: Fig. S8b). These findings were similar to those concerning the 18 PCW kidney, suggesting that the aforementioned signaling effects persisted throughout the kidney development process.

## Discussion

Embryonic renal development is a sophisticated process that includes mesenchymal–epithelial transdifferentiation, progenitor cell self-renewal, and differentiation, branching morphogenesis, and cell–extracellular matrix interactions [2]. scRNA-seq has been widely utilized to explore undetected cell subsets in the developing kidney. However, the cell heterogeneity caused by different spatial regions and the potential effects of spatial cell orientation on the transcriptional expression and biological functions have remained unexplored. In the present study, we integrated ST and scRNA data to depict the spatial distribution of cell subpopulations in the fetal kidney. Additionally, we focused our research efforts on examining the heterogeneity of cell subsets in different cortical and medullary depths, and revealing the impact of spatial signaling transduction on embryonic kidney development.

Energy metabolism is tightly regulated during fetal development. Undifferentiated progenitor and stem cells with highly proliferative potential depend on different combinations of OXPHOS, glycolysis, pentose phosphate pathway, and respiratory chains for proliferation and differentiation [39]. While providing the energy requirements over developmental time, these energy metabolisms also governed developmental cell fate decisions and tissue homeostasis by regulating various cellular signalings such as Wnt, FGF, and PI3K–Akt. The characteristics of cellular energy metabolism in the embryonic kidney have rarely been reported. We found that highly proliferative PTA cells in the deeper cortical region showed an increased dependence on mitochondrial OXPHOS and glycolysis. Although the presence of glycolysis in embryonic stem cells has implicated glycolysis as the preferred pathway of proliferating cells [40], our findings suggested that glycolysis co-existed with mitochondrial OXPHOS in the proliferation of PTA cells. It was reported that mitochondrial respiration generated more ATP than glycolysis, whereas glycolysis provided phospholipids for cytomembrane synthesis, ribose for nucleotide synthesis, and the driving force for chromatin accessibility alterations required for cell proliferation [41, 42]. Compared to PTA cells, NPCs exhibited an inactive energy metabolism in the 18 PCW kidney. Indeed, data from developing mouse kidneys supported that the energy biosynthesis of NPCs declined with the developmental age [43, 44]. Overall, OXPHOS and glycolysis played an important role in maintaining the self-renewing capability of the highly proliferative PTA cells, which also implied that the developmental centre in the 18 PCW kidney has been shifting from the NPC (the outer region) to PTA (the inner region). In addition, we observed that NADH respiratory chain, glycolysis, and OXPHOS were more pronounced in the inner medullary region than in the outer medulla. Unlike PTA, these increased energy metabolic requirements in the deeper medullary region did not correlate with the cell proliferative potential. We speculated that the upregulated expression of OXPHOS-related genes was an efficient pathway to provide ATP for biological processes associated with energy expenditure, such as osmolyte content adjustment, protein metabolism and synthesis, and the maintenance of Na^+^/K^+^ ATPase activity for the control of intracellular sodium concentration [45]. However, elucidating the biological significance of any particular variation still needs additional research evidence.

In the adult kidney, extraordinary osmotic stress poses survival challenges to the cells located in the inner medullary regions, where interstitial fluid normally contains lethally high NaCl and urea levels. The fetal kidney has begun to secrete urine at about 13 PCW, but the gene expression signature of fetal kidney epithelial cells under hyperosmolarity conditions has not attracted much scientific attention. By analyzing the gene sets in different depths of the medullary regions, we revealed that the upregulation of FXYD2, *TonEBP/OREBP*, and *MUPP1/CLDN*4 was essential for cellular viability maintenance. We detected overexpression of FXYD2, a member of the FXYD family of transmembrane proteins that modulates the activity of the γ-subunit of the renal Na/K-ATPase, in the deeper medullary region, which enabled the cell to maintain its source of energy by increasing the apparent affinity for ATP to meet the osmotic stress challenge [46]. Additionally, the osmoprotective transcription factor *TonEBP/OREBP* could potentially translocate from the cytoplasm to the nucleus, where it enhanced the transcription of the genes that generated inert osmolytes (sorbitol, betaine, inositol, and taurine) in response to hypertonicity [20]. We also considered that *MUPP1*/*CLDN4* upregulation was critically important for maintaining the tight junction phenotype of the epithelial sodium channel in the inner medulla. While this may not be of crucial importance to cell viability, it is of considerable significance for sodium balance control [21]. These processes may reflect the intrinsic potential of inner medullary cells to cope with the gradually enhanced osmotic pressure during kidney development, with the aim of preserving the cell vitality and functional integrity.

The nephrogenic niche is critical to the initiation of nephrons and development. Mugford’s study revealed for the first time the heterogeneity in the nephrogenic niche and accidentally identified three distinct compartments in the CM that included the inner capping mesenchyme, the outer capping mesenchyme, and the induced mesenchyme [47]. These nephron progenitor subpopulations with similar gene expression profiles were responsible for distinct biological functions and developmental processes, such as self-renewal, differentiation, and proliferation. The understanding of NPCs in previous studies was based entirely on independent nephrogenic niche structures of the rat model, which failed to embody spatial location information of NPCs in the entire kidney. In the data set presented here, we found that self-renewal NPC cells were located on the cortical surface, whereas NPC-derived PTA cells dominated in the deeper region of the kidney cortex, suggesting that differentiation processes are more likely to occur in the deep cortex. However, we could not distinguish the spatial location of different NPC subpopulations due to the relatively low resolution of the ST-seq.

To better understand the gene modules in the nephrogenic niche, we employed WGCNA analyses, which provided the first insights into the regulatory networks centered on *ETV4/ETV5* and *GDNF*. The transcription factors Etv4 and Etv5 have been shown to be the primary downstream signals for Ret and GDNF-mediated renal branching [29]. Notably, we found that the cycle-regulation gene *CDK1* and some differentiation-related transcription factors such as *FOXD1* and *HOXC4* were also involved in this regulatory process. Cellular communication is also an indispensable regulatory process in kidney development. We highlighted the key role of Wnt, FGF, and Notch pathways in controlling the nephron progenitor cell branching morphogenesis and cell self-renewal among the complex cell communication signaling pathways. *FGF/FGFR* interaction was previously confirmed to induce and correctly position the UB outgrowth in Gdnf–/– mice, indicating that FGF signaling might act in parallel with *GDNF/Ret* to initiate and maintain UB branching morphogenesis [35]. Although Wnt signals (*Wnt7b* and *Wnt9b*) were verified to be essential for the regulation of epithelial cell division and interstitial cell movements, their roles in the induction and development of UB epithelium branching remain unelucidated [36, 37]. Notch signaling (*Notch1* and *Notch2*) is required to establish and maintain the proximal cell fates along the nephron [38]. Most importantly, these pathways exerted persistent effects during fetal kidney development.

There are some limitations of our research. First, the capacity of ST in identifying the cell subpopulations with small numbers in the 9 PCW kidney is insufficient, limiting a comprehensive understanding of specific regions, although this drawback can be improved by cell-type deconvolution analysis. In addition, we also acknowledge that the present study falls short of establishing a dynamic spatial development program of the human fetal kidney due to the lack of the entire embryonic kidney samples at different developmental ages.

In conclusion, the integration of spatial transcriptomics with scRNA data in this investigation enabled the identification of a comprehensive spatial gene expression profile and specific functions in an individual kidney region and provided an understanding of the variations in the spatiotemporal gene landscape, cell differentiation, and cell communication during human kidney development. The findings of the present study are expected to facilitate future research on regenerative medicine and kidney organoid cultivation.

## Supplementary Information

**Additional file 1: Figure S1.** scRNA-seq analysis of two human embryonic kidneys from 9 and 18 post-conception weeks. (a) Schematic of the experimental design and analysis. (b) Distribution of the number of transcripts and genes detected per spot at 9 and 18 PCW kidneys. Blue dashed lines indicate mean values, while the red dashed lines indicate the standard deviation. (c) Dimensionality reduction and clustering of the scRNA-seq data of the 9 and 18 PCW kidneys based on UMAP. (d) Quality control of the scRNA-seq data. ScRNA-seq dataset of fetal kidneys at 9 and 18 weeks post-conception are downloaded online (accession number: GSE114530). (e) Annotation of the cell types identified by spatial transcriptomics data based on the specific cell-type marker genes known from the literature. ST-seq, spatial transcriptomics sequencing; scRNA-seq, single-cell RNA sequencing; PCW, post-conception weeks; ICs, interstitial cells; PTA, pretubular aggregate; UBCD, ureteric bud/collecting duct; SSBmd/DTLH, distal tubule/loop of Henle and s-shaped body medial precursor cell; NPC, nephron progenitor cell; SSBpr, s-shaped body proximal precursor cell; ErprT, early proximal tubule. PETE, Pelvic segment transitional epithelium; Mes, mesangial cell; Pod, podocyte. **Figure S2.** Cell-type deconvolution of the kidney tissues from 9 and 18 weeks post-conception. (a) Spatial location and clustering of ST-seq spots. (b) The proportion of signatures of different scRNA-seq cell types in each ST spot of the 18 PCW kidney tissue. Each pie chart represents the contribution of scRNA-seq cell types to the transcriptomic signature of each ST spot. Only cell types that contribute at least 10% to the spot signature are shown. (c) Up: multimodal intersection analysis (MIA) of scRNA-seq-identified cell types and ST-defined clusters. * represents the closest relationship between scRNA-identified cell types and the ST-defined clusters (P < 0.01). Down: the spatial mapping of scRNA-seq cell subsets performed by MIA. (d) The proportion of each scRNA-seq cell subpopulation arising from each ST spot in the 9 and 18 PCW kidneys. (e) Fraction of total signature of each cell type present in the cortex, medulla, and pelvis in the 18 PCW kidney. (f) Fraction of total signature of each cell type present in the cortex, medulla, and pelvis in the adult human kidney (Ricardo et al.). **Figure S3.** Energy metabolism features of the renal cortex and medulla. Heatmaps displaying the expression levels of (a) OXPHOS-related genes and (b) glycolysis-related genes in scRNA-identified cell types in Hochane’s study. (c) G2/M scores and mean expression levels of proliferation markers (Z-scores) per scRNA-identified cell subpopulation. This image is from Hochane’s study. (d) The distribution feature of the dominated cell types in the cortical region of the 18 PCW kidney. (e) The distribution feature of the dominated cell types in the medullary region of the 18 PCW kidney. **Figure S4.** The biological function of the 16 distinct co-expressed patterns obtained from weighted gene co-expression network analysis. Left, diagrammatic drawing showing the relevance between the 16 modules and corresponding biological function. Scale bar: red represents a strong correlation, and gray indicates that the change in biological function did not reach the P-value threshold. Right, identification of biological function based on the differentially expressed genes in each module. **Figure S5.** Expression levels of M5 network genes for each (a) ST-seq and (b) scRNA-seq cell subpopulation. **Figure S6.** The expression of ribosome-related genes in NPC and PTA clusters identified in Hochane’s study. The structure chart on the top left corner shows the spatial location of the NPC and PTA clusters. Violin plots showing the expression of ribosome-related genes in NPC and PTA clusters. **Figure S7.** Development of the nephric duct. (a) Schematic diagram showing the outgrowth of the UB and its regulatory network. UMPA showing the expression of regulatory genes controlling UB development in the 15 spatial cell clusters. (b) Matching plots show the significant signaling transduction regulation between NPC, PTA, and UB cells (score >0.5 and a P <10^−4^). pink represents the ligand, and blue represents the receptor in the ligand-receptor pairs.

**Additional file 2:** **Table S1.** Quality control of ST-seq date from 9 and 18 PCW kidneys.

**Additional file 3: Table S2. **The proportion of signatures of different scRNA-seq cell types in each ST spot of the 9 and 18 PCW kidney tissues.

**Additional file 4: Table S3. **The biological process of each ST-seq cluster.

## Data Availability

ScRNA-seq dataset of fetal kidneys at 9 and 18 post-conception weeks are available at GEO online database (Accession Number: GSE114530).
