# Supplementary material for: Integrating spatial transcriptomics with single-cell transcriptomics reveals a spatiotemporal gene landscape of the human developing kidney
Source: Cell Biosci. 2022 Jun 3;12:80. doi: 10.1186/s13578-022-00801-x (PMC9164720; doi:10.1186/s13578-022-00801-x)
Supplement: Supplementary file 1 — Additional file 1: Figure S1. scRNA-seq analysis of two human embryonic kidneys from 9 and 18 post-conception weeks. (a) Schematic of the experimental design and analysis. (b) Distribution of the number of transcripts and genes detected per spot at 9 and 18 PCW kidneys. Blue dashed lines indicate mean values, while the red dashed lines indicate the standard deviation. (c) Dimensionality reduction and clustering of the scRNA-seq data of the 9 and 18 PCW kidneys based on UMAP. (d) Quality control of the scRNA-seq data. ScRNA-seq dataset of fetal kidneys at 9 and 18 weeks post-conception are downloaded online (accession number: GSE114530). (e) Annotation of the cell types identified by spatial transcriptomics data based on the specific cell-type marker genes known from the literature. ST-seq, spatial transcriptomics sequencing; scRNA-seq, single-cell RNA sequencing; PCW, post-conception weeks; ICs, interstitial cells; PTA, pretubular aggregate; UBCD, ureteric bud/collecting duct; SSBmd/DTLH, distal tubule/loop of Henle and s-shaped body medial precursor cell; NPC, nephron progenitor cell; SSBpr, s-shaped body proximal precursor cell; ErprT, early proximal tubule. PETE, Pelvic segment transitional epithelium; Mes, mesangial cell; Pod, podocyte. Figure S2. Cell-type deconvolution of the kidney tissues from 9 and 18 weeks post-conception. (a) Spatial location and clustering of ST-seq spots. (b) The proportion of signatures of different scRNA-seq cell types in each ST spot of the 18 PCW kidney tissue. Each pie chart represents the contribution of scRNA-seq cell types to the transcriptomic signature of each ST spot. Only cell types that contribute at least 10% to the spot signature are shown. (c) Up: multimodal intersection analysis (MIA) of scRNA-seq-identified cell types and ST-defined clusters. * represents the closest relationship between scRNA-identified cell types and the ST-defined clusters (P < 0.01). Down: the spatial mapping of scRNA-seq cell subsets performed by [file 13578_2022_801_MOESM1_ESM.docx]

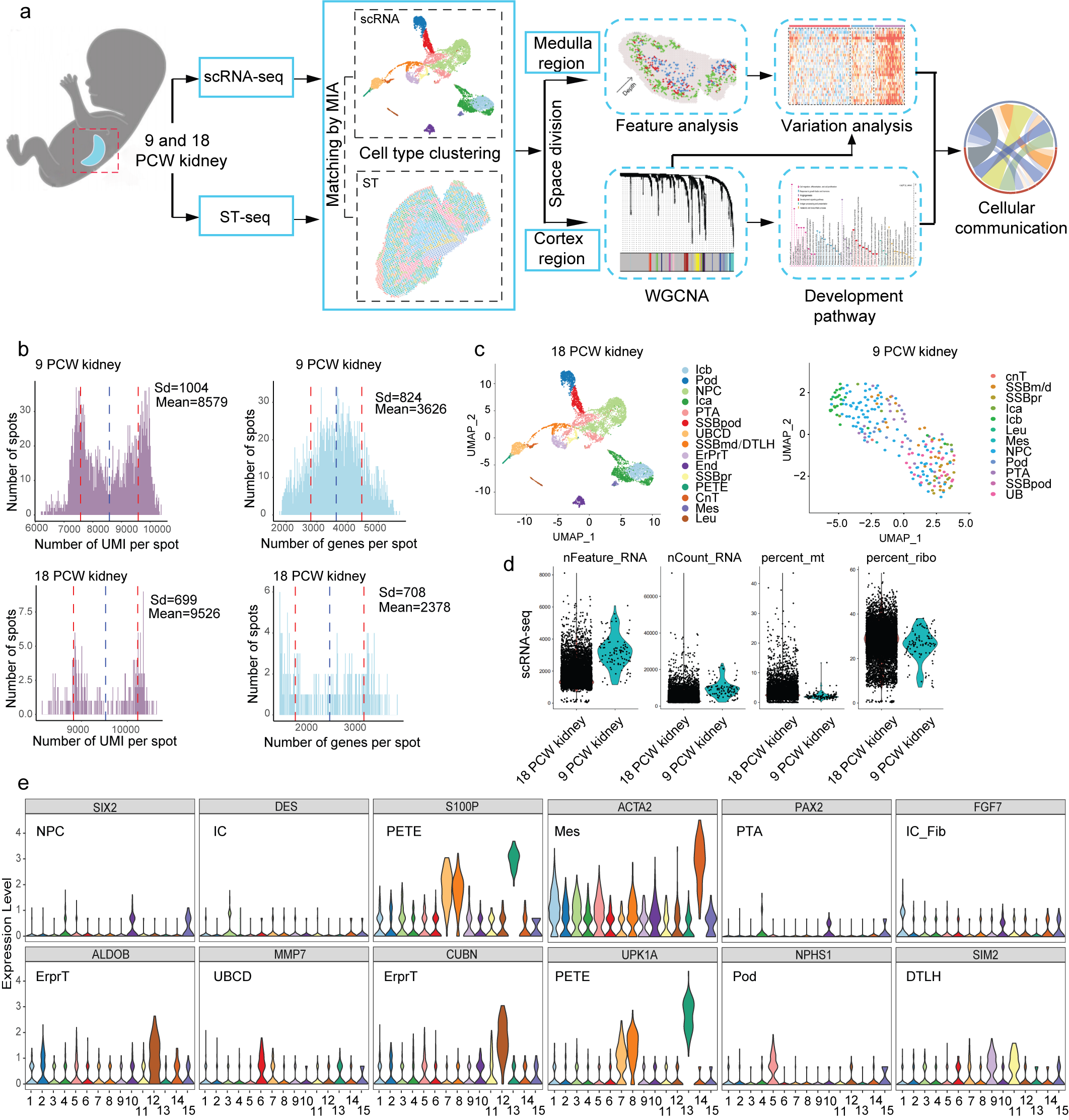


Supplementary Figure 1. scRNA-seq analysis of two human embryonic kidneys from 9 and 18 weeks postconception. (a) Schematic of the experimental design and analysis. (b) Distribution of the number of transcripts and genes detected per spot at 9 and 18 PCW kidneys. Blue dashed lines indicate mean values, while the red dashed lines indicate the standard deviation. (c) Dimensionality reduction and clustering of the scRNA-seq data of the 9 and 18 PCW kidneys based on UMAP. (d) Quality control of the scRNA-seq data. ScRNA-seq dataset of fetal kidneys at 9 and 18 weeks post-conception are downloaded online (accession number: GSE114530). (e) Annotation of the cell types identified by spatial transcriptomics data based on the specific cell-type marker genes known from the literature. ST-seq, spatial transcriptomics sequencing; scRNA-seq, single-cell RNA sequcing; PCW, post-conception weeks; ICs, interstitial cells; PTA, pretubular aggregate; UBCD, ureteric bud/collecting duct; SSBmd/DTLH, distal tubule/loop of Henle and s-shaped body medial precursor cell; NPC, nephron progenitor cell; SSBpr, s-shaped body proximal precursor cell; ErprT, early proximal tubule. PETE, Pelvic segment transitional epithelium; Mes, mesangial cell; Pod, podocyte.


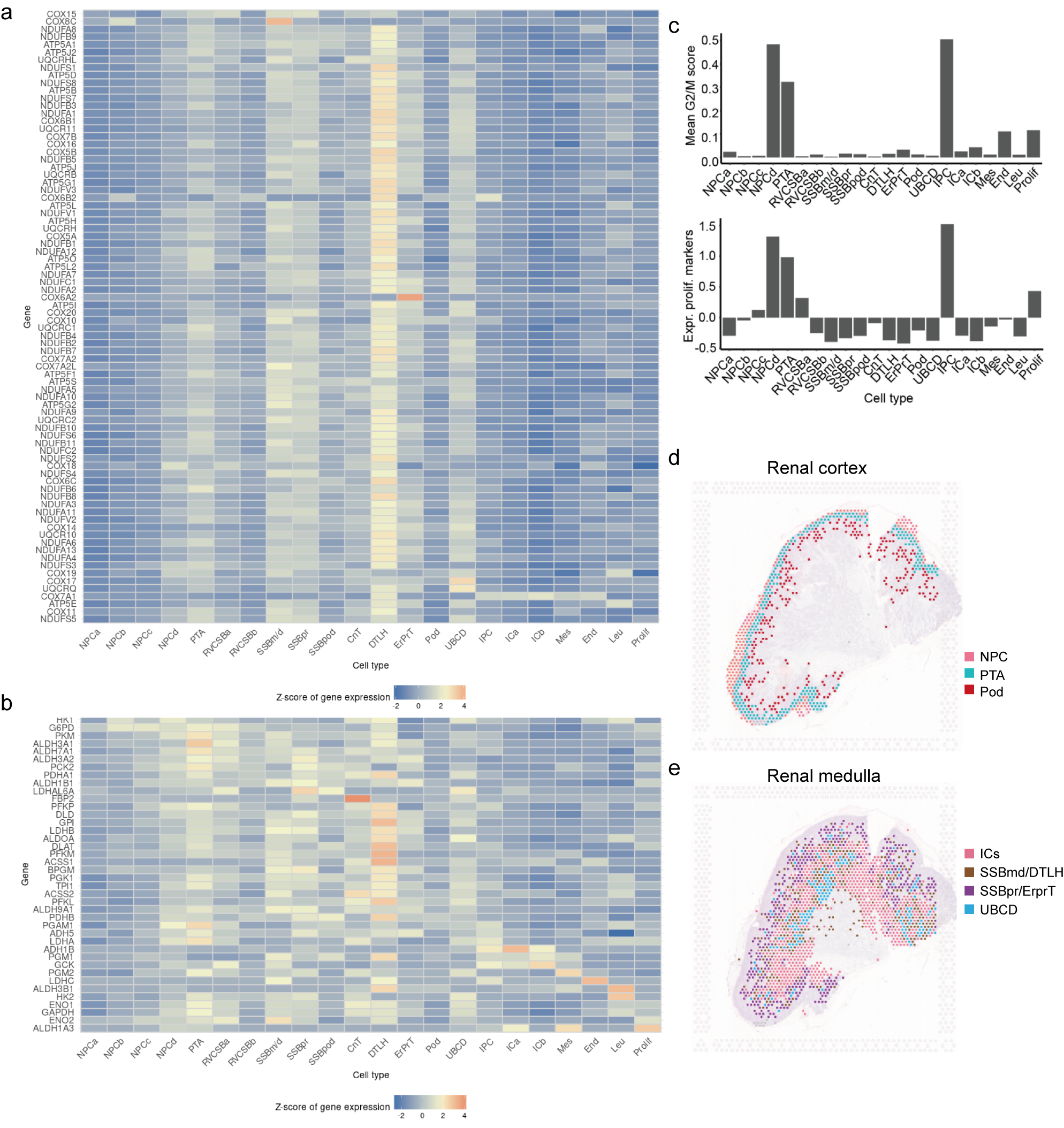


Supplementary Figure 2. Energy metabolism demands of the renal cortex and medulla. Heatmaps displaying the expression levels of (a) OXPHOS-related genes and (b) glycolysis-related genes in scRNA-identified cell types in Hochane’s study. (c) G2/M scores and mean expression levels of proliferation markers (Z-scores) per scRNA-identified cell subpopulation. This image is from Hochane’s study. (d) The distribution feature of the dominated cell types in the cortical region of the 18 PCW kidney. (e) The distribution feature of the dominated cell types in the medullary region of the 18 PCW kidney.


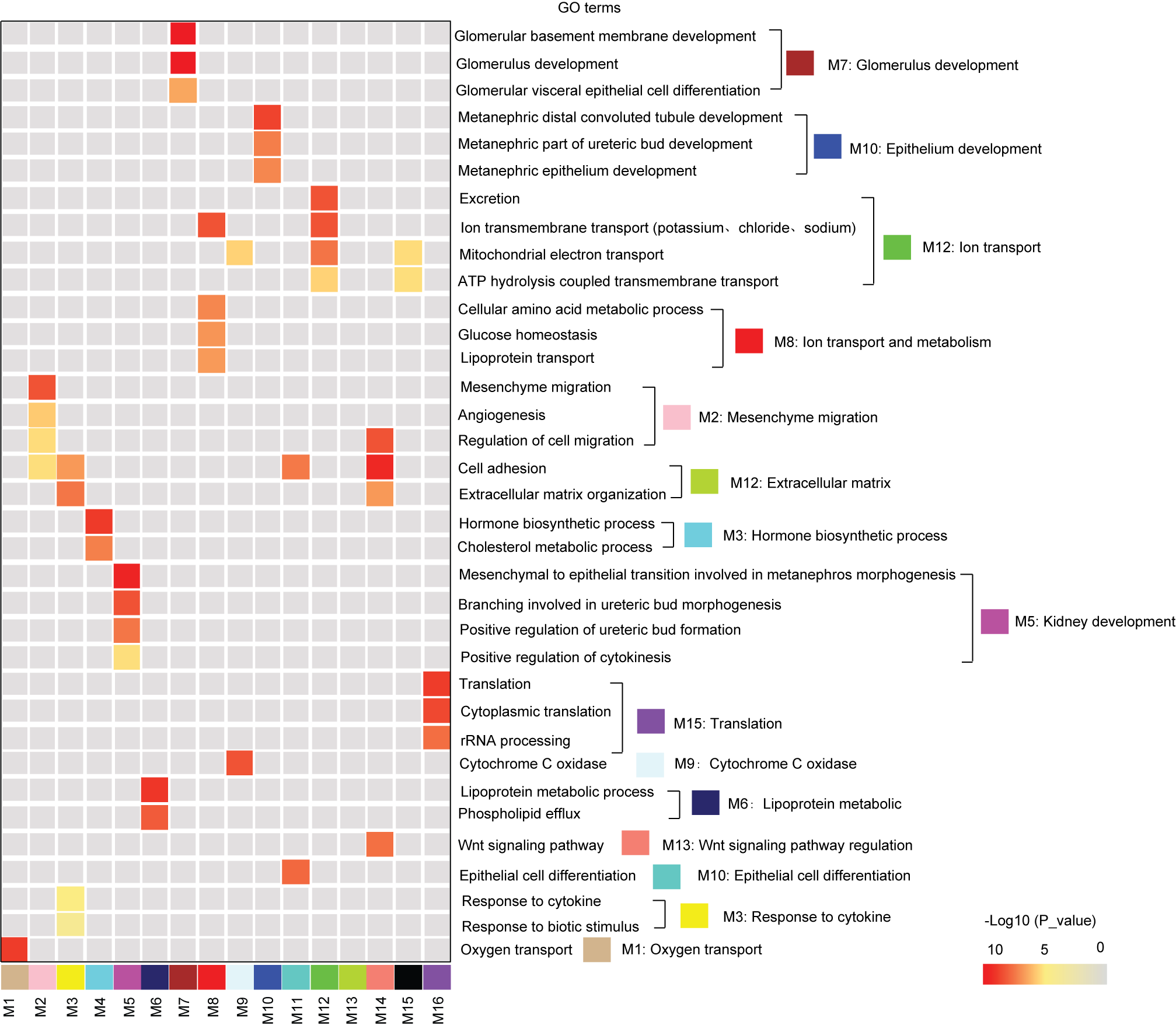


Supplementary Figure 3. The biological function of the 16 distinct co-expressed patterns obtained from weighted gene co-expression network analysis. Left, diagrammatic drawing showing the relevance between the 16 modules and corresponding biological function. scale bar: red represents a strong correlation, and gray indicates that the change in biological function did not reach the P- value threshold. Right, identification of biological function based on the differentially expressed genes in each module.


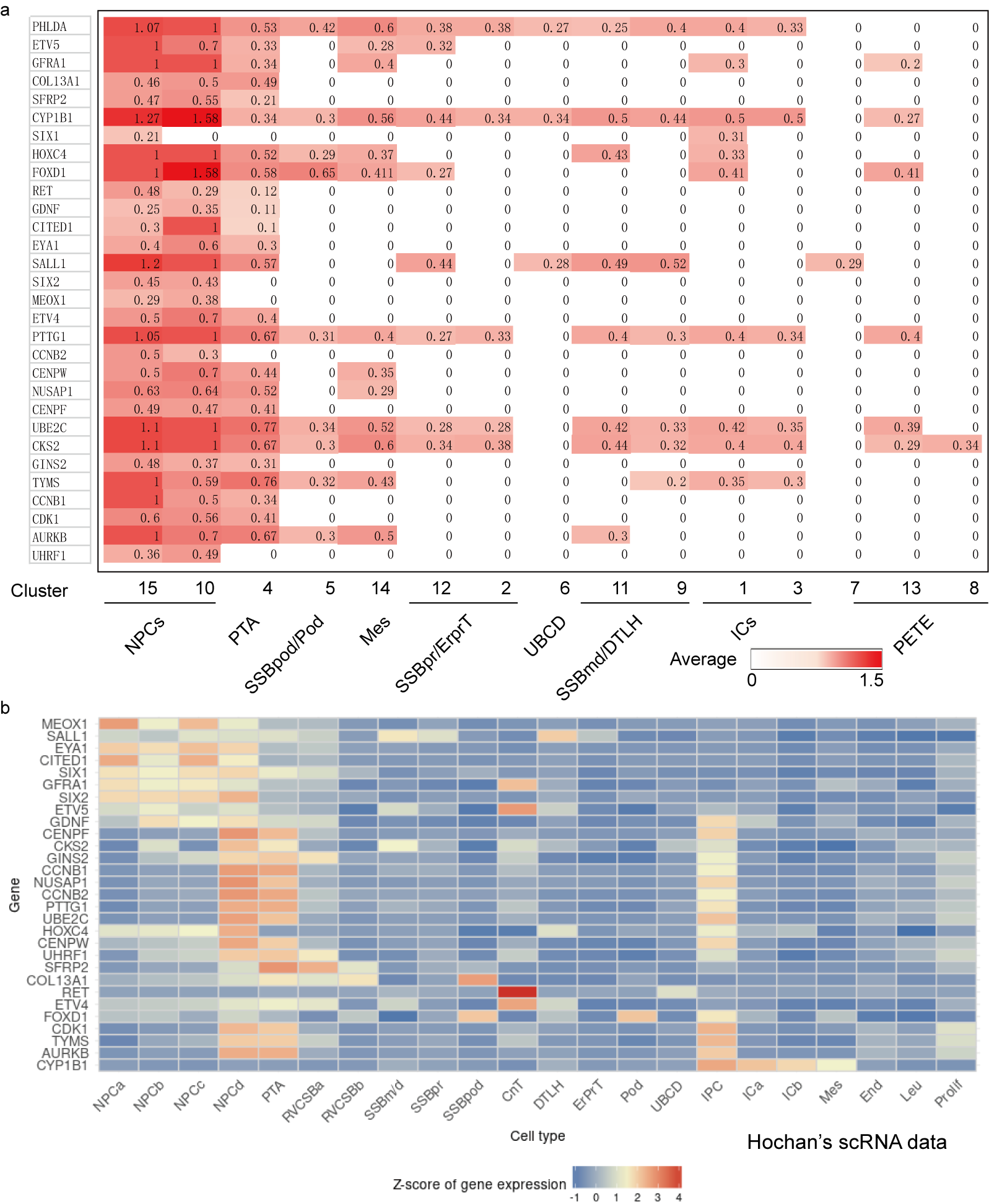


Supplementary Figure 4. Expression levels of M5 network genes for each (a) ST-seq and (b) scRNA-seq cell subpopulation.


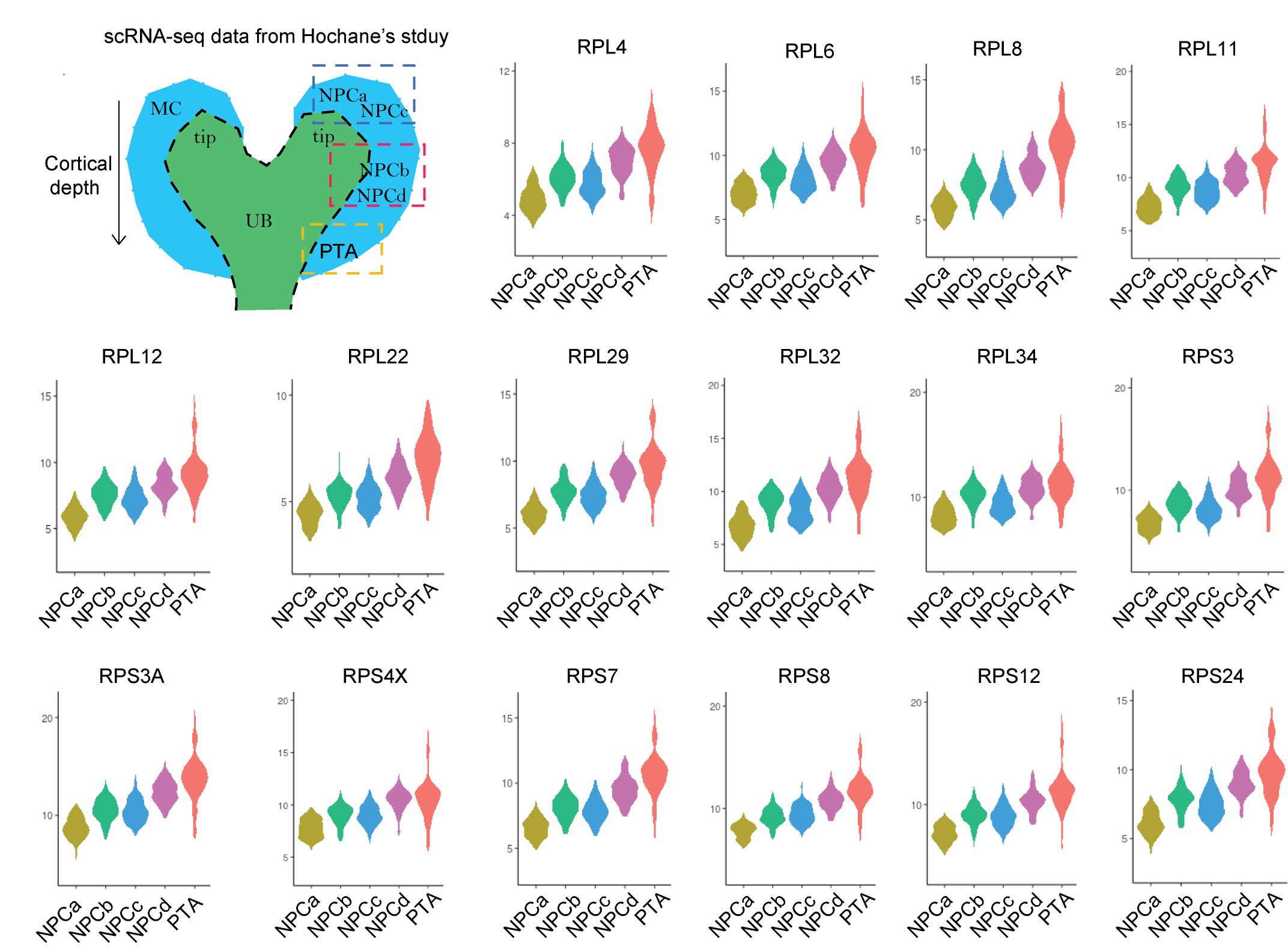


Supplementary Figure 5. The expression of ribosome-related genes in NPC and PTA clusters identified in Hochane’s study. The structure chart on the top left corner showing the spatial location of the NPC and PTA clusters. Violin plots showing the expression of ribosome-related genes in NPC and PTA clusters.


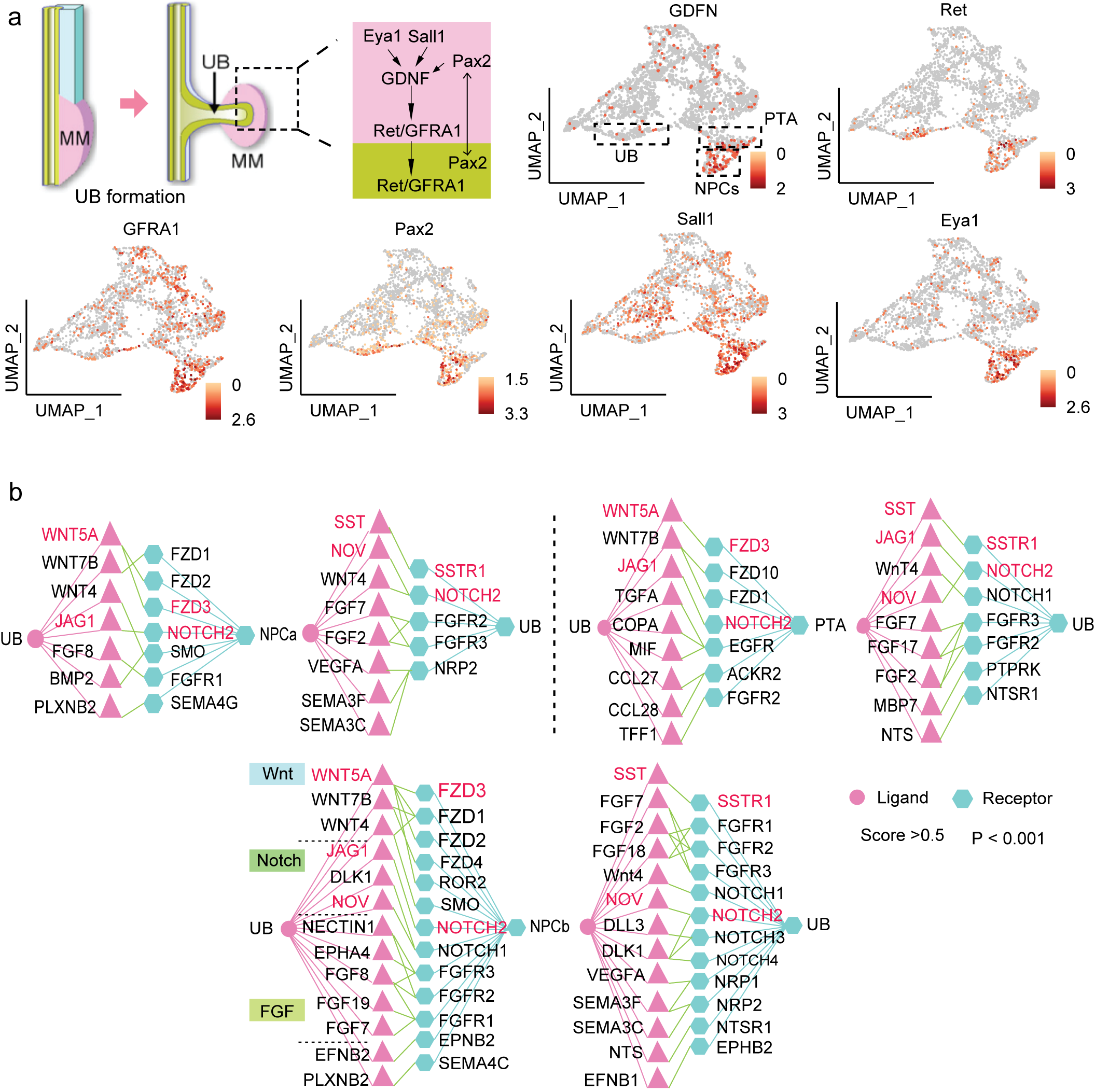


Supplementary Figure 6. Development of nephric duct. (a) Schematic diagram showing the outgrowth of the UB and its regulatory network. UMPA showing the expression of regulatory genes controlling UB development in the 15 spatial cell clusters. (b) Matching plots showing the significant signaling transduction regulation between NPC, PTA and UB cells (score >0.5 and a P <10^-4^). pink represents the ligand, and blue represents receptor in the ligand-receptor pairs.


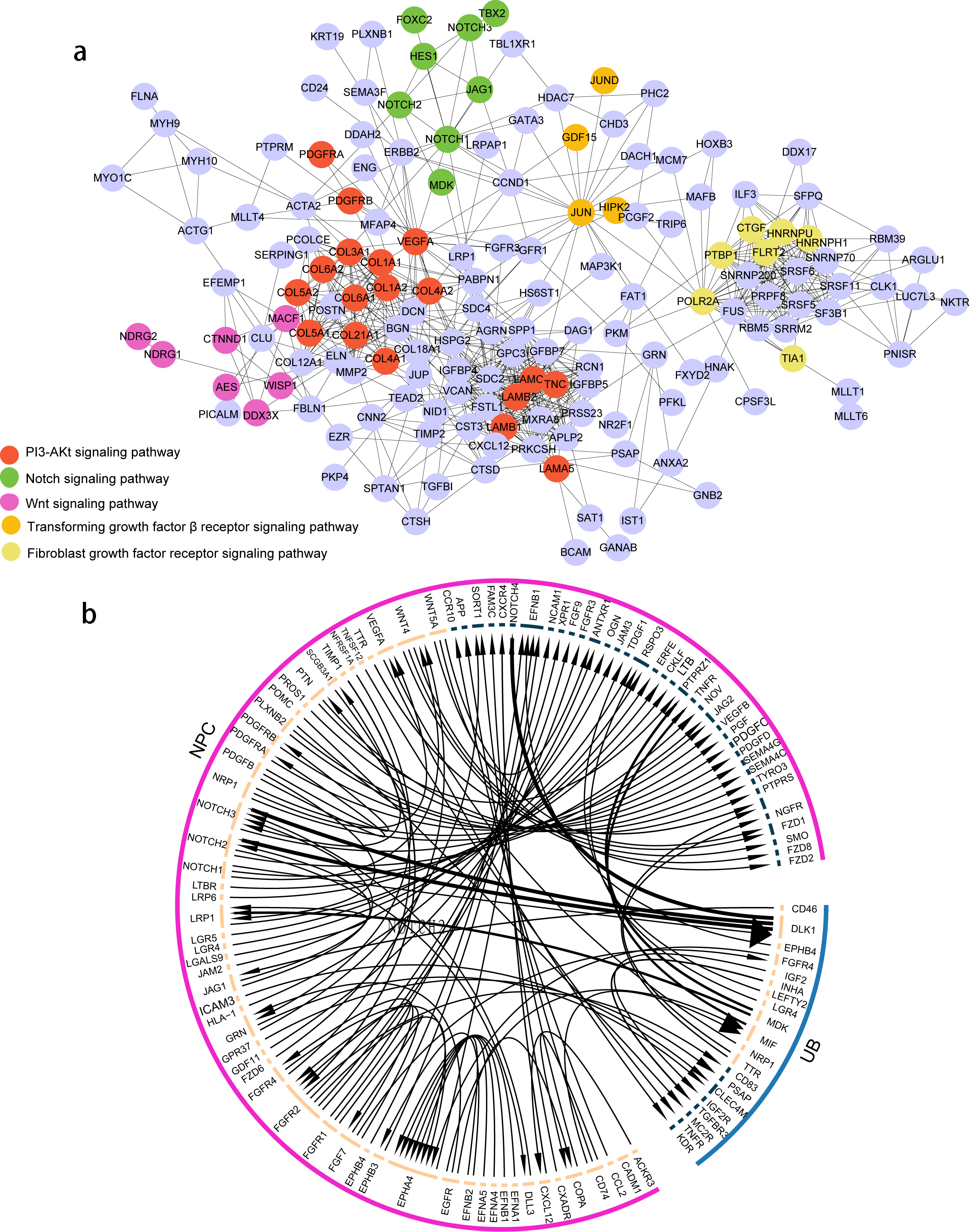


Supplementary Figure 7. Maturation pathways and signaling in the developing kidneys. (a) Protein-protein network showing the correlation of the 366 up-regulated genes during NPC development (from the 8 PCW to 18 PCW kidneys). Genes involved in the common developmental pathways are marked with the same colors. Blue represents genes represent genes that have no significant pathways enrichment. (b) Cellular communication between NPC cluster and UB cluster in the 9 PCW kidney.
